# Biomaterials Functionalized with Inflammasome Inhibitors—Premises and Perspectives

**DOI:** 10.3390/jfb15020032

**Published:** 2024-01-28

**Authors:** Norina Vinţeler, Claudia Nicoleta Feurdean, Regina Petkes, Reka Barabas, Bianca Adina Boşca, Alexandrina Muntean, Dana Feștilă, Aranka Ilea

**Affiliations:** 1 Department of Oral Rehabilitation, Faculty of Dentistry, “Iuliu Hațieganu” University of Medicine and Pharmacy, 400012 Cluj-Napoca, Romania; norina.vinteler@elearn.umfcluj.ro; 2 Department of Chemistry and Chemical Engineering of Hungarian Line of Study, Faculty of Chemistry and Chemical Engineering, Babeș-Bolyai University, 400028 Cluj-Napoca, Romania; petkesregina@yahoo.com (R.P.); reka.barabas@ubbcluj.ro (R.B.); 3 Department of Histology, Faculty of Medicine, “Iuliu Hațieganu” University of Medicine and Pharmacy, 400012 Cluj-Napoca, Romania; bianca.bosca@umfcluj.ro; 4 Department of Paediatric, Faculty of Dentistry, “Iuliu Hațieganu” University of Medicine and Pharmacy, Cluj-Napoca 400012, Romania; alexandrina.muntean@umfcluj.ro; 5 Department of Orthodontics, Faculty of Dentistry, “Iuliu Hațieganu” University of Medicine and Pharmacy, Cluj-Napoca 400012, Romania; dana.festila@umfcluj.ro

**Keywords:** inflammation, inflammasome, inhibitors of the inflammasomes, nanomaterials, functionalized biomaterials, periodontitis, general pathology

## Abstract

This review aimed at searching literature for data regarding the inflammasomes’ involvement in the pathogenesis of oral diseases (mainly periodontitis) and general pathologies, including approaches to control inflammasome-related pathogenic mechanisms. The inflammasomes are part of the innate immune response that activates inflammatory caspases by canonical and noncanonical pathways, to control the activity of Gasdermin D. Once an inflammasome is activated, pro-inflammatory cytokines, such as interleukins, are released. Thus, inflammasomes are involved in inflammatory, autoimmune and autoinflammatory diseases. The review also investigated novel therapies based on the use of phytochemicals and pharmaceutical substances for inhibiting inflammasome activity. Pharmaceutical substances can control the inflammasomes by three mechanisms: inhibiting the intracellular signaling pathways (Allopurinol and SS-31), blocking inflammasome components (VX-765, Emricasan and VX-740), and inhibiting cytokines mediated by the inflammasomes (Canakinumab, Anakinra and Rilonacept). Moreover, phytochemicals inhibit the inflammasomes by neutralizing reactive oxygen species. Biomaterials functionalized by the adsorption of therapeutic agents onto different nanomaterials could represent future research directions to facilitate multimodal and sequential treatment in oral pathologies.

## 1. Introduction

Inflammation and inflammasome activation are the main mechanism underlying oral pathologies, particularly periodontitis, and the interrelation between oral and systemic diseases. Periodontitis is a chronic inflammatory disease, caused by pathogenic bacterial species from the dental biofilm, which also trigger the inflammatory immune response of the host. Among periodontal pathogenic species, the most prominent are *Porphyromonas gingivalis* and *Aggregatibacter actinomycetemcomitans*. The presence of inflammatory and immune cells is essential for maintaining periodontal health. Under physiological conditions, various bacterial species that colonize the gingival sulcus live in symbiosis. Once homeostasis is altered, the imbalance between the host’s immune response and the microbiome leads to dysbiosis, with the consequent onset of periodontal disease. The inflammatory process is the first activated defense mechanism against periodontal pathogenic bacteria. The inflammasomes are part of the innate immune response that activates inflammatory caspases. Once an inflammasome is activated, pro-inflammatory cytokines, such as interleukins, are released. Furthermore, when an inflammasome is activated, a mechanism of programmed cell death called pyroptosis is initiated. Recently developed therapeutic approaches for controlling inflammation have proposed inflammasome inhibition by using phytochemicals and pharmaceuticals. Pharmaceutical substances can control inflammasomes by three mechanisms: inhibiting intracellular signaling pathways, blocking inflammasome components, and inhibiting cytokines mediated by the inflammasomes. Additionally, phytochemicals could inhibit inflammasome activation by neutralizing reactive oxygen species. 

Periodontal disease progresses through periodontal tissue breakdown, affecting both the periodontal ligament and the alveolar bone. For bone replacement, the therapeutic guidelines involve complex surgical therapies, such as bone grafts, the use of platelet-rich plasma, or guided tissue regeneration. The use of nanobiomaterials could facilitate tissue healing and improve treatment outcomes. The adsorbtion of pharmaceutical agents on the surface of nanobiomaterials could enable the controlled release at the affected periodontal site. Detailed research of the physico-chemical properties of the phytochemical and pharmaceutical substances with an inhibitory effect on inflammasomes could indicate the optimal method for their adsorption on various nanostructures, in order to be used for clinical applications. These materials have numerous advantages, such as the controlled release of therapeutical agents, as well as disadvantages related to their biodegradation and cytotoxicity. Although nanobiomaterials were intensively studied in various medical and related fields, the transfer of this information to the usual practical applicability has not yet been achieved. Through future research focused on reducing their disadvantages, functionalized nanobiomaterials could become important adjuvants in medical practice. 

This review aimed at searching literature for data regarding the inflammasomes’ involvement in the pathogenesis of oral diseases (mainly periodontitis) and general pathologies, including approaches to control inflammasome-related pathogenic mechanisms. The review also investigated novel therapies based on the use of phytochemicals and pharmaceutical substances for inhibiting the inflammasomes’ activity.

## 2. Literature Review Section

### 2.1. Neutrophils—Activation and Involvement in Periodontal Disease

**Figure 1:**
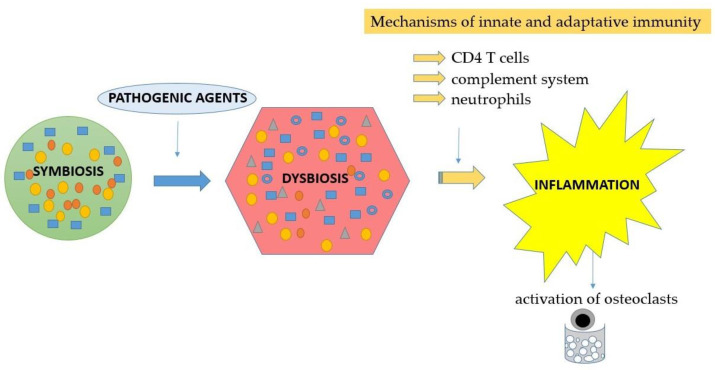
The cascade underlying the transition of the oral microbial communities from symbiosis to dysbiosis, with the onset of inflammation and activation of adaptive immunity, followed by osteoclasts’ differentiation and alveolar bone resorption.

Neutrophils play a major role in periodontal health and disease. The absence or functional deficit of neutrophils induces the onset of severe forms of periodontitis. Thus, functional neutrophils have proven to be essential for periodontal homeostasis. However, the increased number of neutrophils or their hyperreactivity causes imbalanced interactions between the host and the microbiome, which leads to dysbiosis and periodontal breakdown [1]. Dysbiosis results from the disruption of homeostasis between the host’s immune system and the oral microbiome. Thus, microbial communities develop pathogenic mechanisms that overcome the immune response of the host, causing an inflammatory reaction and the progression of periodontal disease. Williams et al. developed a single-cell transcriptome atlas of the dental and gingival tissues designed for identifying the predominant cell populations in health and disease. The analysis of epithelial biopsies from the oral and gingival tissues of patients with periodontitis demonstrated a significant correlation between inflammation and neutrophil activation [2]. Neutrophils represent the most numerous inflammatory cells implicated in innate immunity. Neutrophils exert antimicrobial activity and have the ability to release inflammatory mediators that fight against pathogenic microorganisms. Under physiological conditions, neutrophils’ responses against microbial communities maintain self-limiting inflammation. In the case of dysbiotic microbial attack, neutrophils show intensified activity, leading to an exaggerated inflammatory response, which further results in disease progression. The transition of commensal microbial communities from symbiosis to dysbiosis triggers tissue inflammation by involving mechanisms of both the innate and adaptive immunity [3]. These include the complement system, CD4 T cells and neutrophils, which, through inflammation, promote osteoclasts’ activation and the resorption of alveolar bone [4] (Figure 1).

Neutrophils possess the ability to respond to the attack of microorganisms, which tend to develop survival strategies during disease progression [5]. The hyperreactive and overnumbered neutrophils discharge toxic substances in the mucosal tissues, thus contributing to tissue damage. The role of neutrophils in maintaining oral health has been demonstrated by the association between neutrophilia, neutropenia and neutrophils’ functional deficit and periodontal disease [6]. The antimicrobial functions of neutrophils are based on phagocytosis, degranulation and the extrusion of chromatin DNA, which results in the formation of neutrophil extracellular traps (NETs). Degranulation represents an antimicrobial attack performed by neutrophils, with major implications on the progression of periodontal disease [7]. Neutrophils can be classified into positive and negative peroxidases based on the presence of myeloperoxidase in the contents of the granules. In addition to positive peroxidases, the matrix contains other powerful antimicrobial compounds, such as serine proteases, neutrophil elastase (NE), proteinase 3 (PR3) and cathepsin G. The negative peroxidase contains antimicrobial compounds such as lactoferrin, lysozyme and metalloproteinases (MMPs): gelatinases and collagenases [8]. The degree of neutrophil stimulation determines the hierarchical granular exocytosis. The sequence of neutrophil degranulation takes place inversely to granule formation, during maturation in the bone marrow. The data from the literature of the last two decades have shown that neutrophils are not limited to their antimicrobial capacity in the context of inflammation [9]. Neutrophils perform both antimicrobial and immunomodulatory functions. Neutrophils’ response to microbial attack must be properly regulated, in order to minimize the negative effects on the host tissue. Priming is a reversible high alert state of neutrophils, which activates phagocytosis, degranulation, NET formation and the release of inflammatory mediators [10]. Neutrophil priming is induced by endogenous inflammatory mediators: cytokines, growth factors, chemokines and by lipopolysaccharides (LPS). When neutrophil priming occurs during chronic systemic inflammation, it causes harmful effects [11]. The priming of circulating neutrophils can occur through exposure to low systemic levels of cytokines or chemokines. When these neutrophils reach the gingival sulcus, the secondary stimulation by local microorganisms leads to an exacerbated global response. Thus, neutrophil priming can have both beneficial and harmful effects. Insufficient priming is associated with recurrent infections [12], whereas increased priming leads to chronic inflammatory oral diseases, particularly periodontitis, and systemic diseases, such as rheumatoid arthritis or sepsis [13,14,15]. Circulating neutrophils isolated from patients with periodontitis have shown the increased production of reactive oxygen species (ROS) upon exposure to soluble stimuli, such as phorbol myristate acetate (PMA), or to bacterial stimulation by *Porphyromonas gingivalis*, compared to the response elicited by cells from a healthy control group [14].

**Figure 2:**
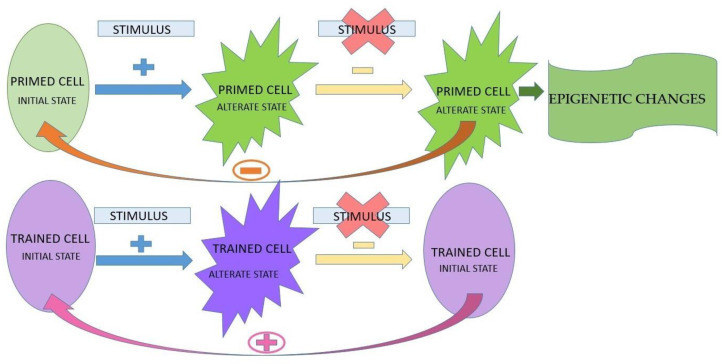
The reaction to a stimulus of primed cells versus trained cells, and the induction of epigenetic changes. 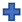 The presence of the stimulus; 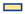 the absence of the stimulus; 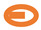 the absence of return to the initial state; 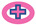 the presence of return to the initial state.

Primed cells with an altered functional state do not return to the initial state, thus improving the inflammatory response in the short term, which is in contrast to trained cells returning to the initial state, which has long-term effects. The changes that persist in primed innate immune cells are called epigenetic changes, and contribute to immunological memory [16] (Figure 2). 

In the case of dysbiosis, the pathogenic bacterial communities develop various survival mechanisms to increase their resistance to the host’s immune reaction; these mechanisms also enable bacteria to benefit from the host’s inflammatory response. Some pathogens can survive the immune attack by taking advantage of molecules derived from neutrophils, while other pathogens survive by developing resistance to the antimicrobial attack [7]. In periodontitis patients, active oral lesions create an inflammatory state, which provides favorable conditions for the growth and development of oral pathogens. 

**Figure 3:**
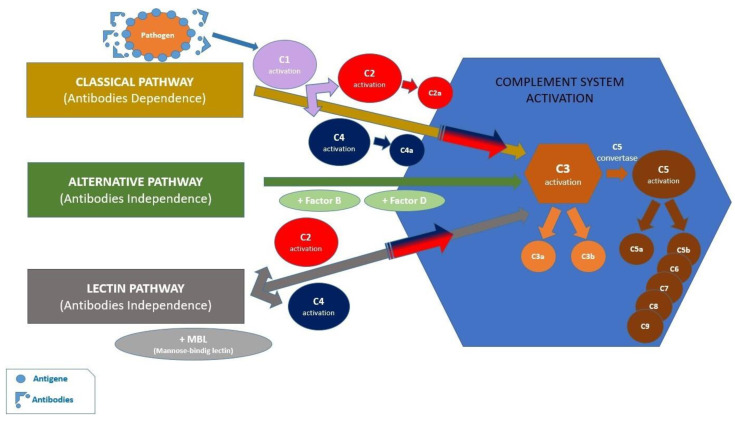
Activation of complement system through three pathways, and their convergence towards the C3 central complement component.

*Porphyromonas gingivalis* is a Gram-negative oral pathogen, a strictly anaerobic and asaccharolytic bacterium, which predominantly populates the gingival sulcus [17]. This pathogen is able to evade the antimicrobial attack of macrophages and neutrophils, but it also benefits from the nutritional substrate created by gingival inflammation. Neutrophils participate in the initial antimicrobial attack by recognizing pathogens due to the recognition receptors (PRRs), the most important of which is the Toll-Like receptor (TRL), capable of recognizing distinct microbial structures [18]. Symbiosis is also maintained by a mechanism involving the innate immune system, with the participation of the complement system comprising approximately 50 types of proteins [19]. The complement system is activated by three pathways: the classical activation, the alternative activation and the lectin pathway. All these activation pathways converge towards the central C3 component [20] (Figure 3). *Porphyromonas gingivalis* interacts with cells of the innate immune system by binding to CD14, which is a crucial coreceptor for TLR2 activation. Thus, the phosphatidylinositol 3-kinase (PI3K) family of enzymes is activated, which acts on β2 integrin in monocytes and neutrophils [21]. *Porphyromonas gingivalis* can also bind to complement receptor 5 (C5aR1) and the C5a ligand. *Porphyromonas gingivalis* expresses cysteine proteases, called gingipains, providing the virulence necessary to evade the antimicrobial response [22]. Gingipains can cleave the C5 component of the complement, leading to the formation of C5a, a strong chemotactic neutrophil molecule, and C5b, which prevents terminal complement activation. According to a recent study, the blockage or genetic absence of the C5aR1 receptor reduces the colonization ability of *Porphyromonas gingivalis* [23].

*Aggregatibacter actinomycetemcomitans* is a Gram-negative, facultative anaerobic and non-motile coccobacillus in the *Pasteurellaceae* family. As an opportunistic oral pathogen, it possesses several virulence factors, responsible for its survival [24]. *Aggregatibacter actinomycetemcomitans* expresses a two-component receptor called QseBC, which enables bacterium activation by the combination of iron and catecholamines (epinephrine) [25]. A recent study demonstrated that neutrophils are a source of catecholamines due to epinephrine stored in the azurophilic granules. Increased iron levels in the microenvironment and epinephrine released from neutrophils promote the growth of *Aggregatibacter actinomycetemcomitans*, which results in the induction of the QseBC operon [26]. 

The “neutrostat” concept defines the homeostatic mechanism of neutrophils’ recruitment and efferocytosis in the tissues. The elimination of apoptotic neutrophils by tissue macrophages prevents secondary necrosis and inhibits IL-23 expression. The inhibition of IL-23 in conjunction with efferocytosis enables further reduction in IL-17 production by CD4 T cells, subsequently controlling the neutrophils’ formation in the bone marrow [27].

Hypercholesterolemia and hyperglycemia are additional factors that alter neutrophils’ production and functionality [28]. Hypercholesterolemia is associated with increased production and mobilization of “primed” circulating neutrophils and their exaggerated response to secondary stimulation [29]. In hyperglycemia, some neutrophil-derived factors can increase ROS production and NET formation [30].

Complement plays key roles in inflammation due to its involvement in neutrophils’ recruitment and optimal functionality. Neutrophils, through specific receptors, respond to complement activation fragments, C3a and C5a [31]. A recent study demonstrated that the inhibition of the C3 fraction blocked periodontal inflammation and reduced bone loss in non-human primates (NHPs) [32].

### 2.2. Activation of the Inflammasome

Inflammation represents a quick and early defense against pathogens. The inflammatory response can be physiological, when induced by the interaction between the host and different stimuli, thus offering rapid protection against the attack [33]. 

Inflammasomes are part of a pathway of the innate immune reaction that activates inflammatory caspases, a family of cysteine proteases [34]. According to a study from 2002, the inflammasome is a multiprotein complex activated by different pathogens, which induces the activation of caspase-1. The term “inflammasome” has been used since 2009, after the publication of the study conducted by Bostanci et al. [35]. Inflammasomes have different names, according to the class of PRR sensor molecule that induces their activation [36]. NLR proteins have been demonstrated to play regulatory roles in the innate immune response [37,38]. 

The most important inflammasomes include NLRP2, NLRP6, NLRC4 and AIM2 [39]. The first discovered inflammasome was NLRP1, which is activated by *Bacillus anthracis* and mediates the activation of caspase-1 [40]. Several studies have demonstrated that the NLRP1 inflammasome is not significantly activated neither by *Porphyromonas gingivalis* [41] nor by *Aggregatibacter actinomycetemcomitans* [42]. The NLRP2 inflammasome inhibits NF-kB activation [43]. The NLRP3 inflammasome can be activated by a number of factors, including LPS, *Staphylococcus aureus*, *Klebsiella pneumoniae* and *influenza virus* [44], but also by uric acid and ATP [45]. The NLRP6 inflammasome is involved in intestinal homeostasis [46], and *Aggregatibacter actinomycetemcomitans* infection reduces its activity [42]. The NLRP7 inflammasome is associated with *Staphylococcus aureus* infection [47], but there is no scientific evidence to demonstrate its link to periodontal disease. The NLRP12 inflammasome inhibits the activation of NF-kB and activates TLR and TNF-α [48]. The NLRC4 inflammasome controls the release of IL-1β and influences NF-kB [49], whereas the NLRC5 inflammasome acts similarly to the NLRP3 inflammasome, by decreasing the activity of caspase-1 and the interleukins IL-1β and IL-18 [50].

The inflammasome, a multi-protein oligomer, plays important roles in inflammatory processes [51]. Inflammasomes are classified into Nucleotide-binding domain leucine-rich repeat (NLR) inflammasomes and PYHIN (pyrin and hematopoietic interferon) inflammasomes [52]. Furthermore, the NLR class is divided into the NBD (nucleotide binding domain) subclass, which is involved in ribonucleotide binding, the regulation of oligomerization and inflammasome assembly, and the LRR (leucine-rich proteins) subclass, which is involved in the recognition of PAMPs and in interprotein interactions [53]. According to the literature, the NLRP3 inflammasome is implicated in caspase-1 activation and the proteolytic processing of pro-IL-1β [39]. Caspases modulate the immune response by cleaving specific cellular substrates [34]. Inflammasome activation causes the release of pro-inflammatory cytokines and initiates a mechanism of programmed cell death called pyroptosis [54]. 

Apoptosis was the first type of cell death described as “programmed cell death”, in which the cell plays an active role in its own destruction [55]. Other mechanisms of cell death are represented by *autophagy* (the consumption of its own components in an organism subjected to starvation) [56], *necroptosis* (a type of programmed necrosis that occurs during caspase inhibition or insufficient caspase activation) [57], *ferroptosis* (programmed cell death dependent on iron and characterized by the accumulation of lipid peroxides) [58], *NETosis* (the process by which extracellular neutrophil traps are formed) [59] and *pyroptosis* (a destruction mechanism mediated by caspases) [60]. 

Pyroptosis was firstly described in 1992 in macrophages infected with *Shigella Species* [61], and later in infections with *Salmonella typhimurium* [62]. This is a process of self-destruction mediated by caspases, which sets in quickly and causes the release of pro-inflammatory cytokines [63]. The name pyroptosis is derived from the Greek words “pyro”, meaning fire, and “ptosis”, which means falling [62]. Pyroptosis also induces a series of morphological and physiological changes associated with inflammatory reactions [60]. Due to the pores in the membrane of pyroptotic cells, the membrane permeability is altered [64]; thus, viruses passed from the intracellular to the extracellular compartment become more susceptible to the immune attack [60]. The subsequent release of pro-inflammatory cytokines from the cytoplasmic granules into the extracellular environment induces cell death, and implicitly an inflammatory response [65]. Contrarily to pyroptosis, apoptosis is a controlled form of cell death that does not result in inflammation or the destruction of adjacent cells [66]. One of the mechanisms for the activation of inflammasomes and caspase-1 is triggered by staphylococcal α-hemolysin, a bacterial toxin produced by *Staphylococcus aureus* [67]. Another mechanism is based on the activation of caspase-1, due to the lower levels of intracellular potassium (K^+^) induced by nigericin, a toxin produced by *Streptomyces hygroscopicus* [68]. The nigericin–K complex, formed by nigericin and K^+^, is transported across the cell membrane and released extracellularly [69]. Lipopolysaccharides are also able to activate caspases and induce gasdermin D (GSDMD) cleavage [33]. 

**Figure 4:**
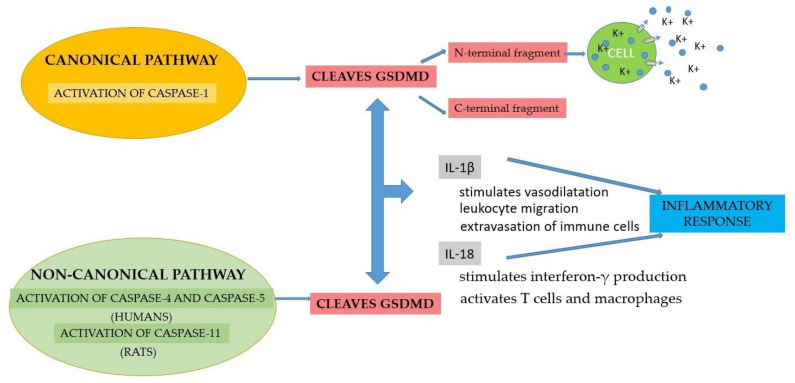
Schematic representation of canonical and non-canonical pyroptosis activation. Both pathways lead to GSDMD cleavage, followed by release of interleukins 1β (IL-1β) and 18 (IL-18), and the subsequent initiation of inflammatory response.

Pyroptosis is a process of cellular self-destruction mediated by caspases. When cells are stimulated by pathogens, inflammasome formation is triggered. Pyroptosis is mediated through caspase-1 activation by the NLRP3 inflammasome. Caspase-1 cleaves GSDMD, causing cell membrane cleavage, a process termed canonical inflammasome activation [63]. In contrast, the non-canonical activation of pyroptosis is induced by the activation of caspase-4 and -5 in humans and caspase-11 in rats, which will cause GSDMD cleavage. Although pyroptosis mechanisms can be activated by different signaling pathways, they all lead to the activation of GSDMD. Subsequently, cytoplasmic molecules such as interleukins 1β (IL-1β) and 18 (IL-18) are released by GSDMD and trigger an inflammatory response [70] (Figure 4). 

Pyroptosis is mediated by the activation of caspases [71]. Even though pyroptosis is a protective mechanism against pathogens, its overactivation causes damage to adjacent tissues [72]. The canonical pathway is represented by the detection of the pathogenic signal, followed by the formation of inflammasomes, due to the immune cells of the innate immunity, which activate capsase-1. The non-canonical inflammasome pathway requires the presence of caspases 4 and 5, which will trigger pyroptosis. Caspase-1 cleaves GSDMD into two fragments: the N-terminal fragment and the C-terminal fragment. The N-terminal fragment causes the formation of small-sized pores (10–15 nm) in the cell membrane, which allow for the efflux of the cytoplasmic content and the increased efflux of K^+^, which activates caspase-1, through the NLRP3 inflammasome [73]. Pyroptosis also induces the release of cytokines, such as IL-1β and IL-18. IL-1β stimulates vasodilatation, leukocyte migration and the extravasation of immune cells, and IL-18 promotes interferon-γ production and activates T cells and macrophages [60] (Figure 4).

IL-1 consists of two subunits: IL-1α induces the production of inflammatory mediators, the differentiation of osteoclasts, and causes the destruction of alveolar bone and periodontal connective tissue, whereas IL-1β is involved in the progression of periodontal disease [74]. IL-18 is produced in an inactive form and is activated by caspase-1, with the implication of the activation of the inflammasome. The inflammasome consists of NLR, which is a leucine-rich receptor [65]. Several types of NLRs are involved in inflammasome activation, the most common of which is NLRP3 [75]. 

A number of activators of the NLRP3 inflammasome have been discovered, such as silicon particles, asbestos, cholesterol crystals, bacterial toxins, K^+^ efflux, cathepsin B, ribosomal mRNA, bacterial ATP, oxidized mitochondrial DNA, ROS, blocking function ribosomes, bacterial RNA, lipopeptides, guanylate-binding protein 5 and mycoplasmas [76], extracellular ATP and uric acid crystals. Inflammasome activation requires the participation of a sensor (NLRP3), which fuses with an adapter (apoptosis-associated speck-like protein—ASC adapter), resulting in the formation of a filamentous structure that provides a platform for Caspase-1 cleavage [77]. The NOD3 inflammasome receptor protein (NLRP3) is activated by a series of microbial and metabolic signals. A priming step involving Toll-like receptors (TLRs) is required, causing the increase of IL-1 and the inflammasome components Caspase-1 and NLRP3. Afterwards, the components of the inflammasome gather in the cytoplasm and Caspase-1 is cleaved [78]. Recent studies have demonstrated that priming induces the synthesis of mitochondrial DNA and the mitochondrial enzyme deoxyribonucleotide kinase, which activates the NLRP3 inflammasome [79].

### 2.3. Implications of the Inflammasome in Periodontal Disease

Inflammasome activation within periodontal tissues is an important mechanism in the pathogenesis of periodontal disease.

Periodontal disease is infectious chronic inflammation leading to the destruction of the supporting periodontal tissues [33]. Periodontitis is initiated by the invasion of Gram-negative anaerobic bacteria, such as *Porphyromonas gingivalis*, *Aggregatibacter actinomycetemcomitans*, *Treponema denticola*, *Fusobacterium nucleatum* and *Tannerella forsythia* [63]. Numerous studies have reported that inflammasome levels [35], NLRP3 expression [80] and caspase and IL-18 tissue concentrations are increased in periodontal disease compared to healthy tissues [81]. There is contradictory data regarding the influence of *Porphyromonas gingivalis* on inflammasomes; some studies have indicated inflammasome activation and IL-1β production, whereas other studies have reported inflammasome inhibition [76].

Pyroptosis represents one of the pathways leading to the exacerbation of periodontal inflammation. According to current studies, virulence factors present in the affected periodontal tissues induce inflammasome activation and tissue destruction through caspase activation, the cleavage of gasdermin D and the secretion of interleukins IL-1β and IL-18 [55] (Figure 4). The involvement of IL-1 in periodontal disease was mentioned for the first time in 1985 [82]; then, in 1991, the role of the IL-1β subunit was demonstrated [83]. According to a study carried out in 1992, IL-1 levels were increased in subjects with gingivitis [84], and IL-1 could be considered an early marker of periodontal inflammation. A study performed in 1997 demonstrated that IL-1 and TNF play an important role in the pathogenesis of periodontal disease [85]. In 2009, it was demonstrated that NLRP3 and NLRP2 presented increased levels in affected periodontal tissues, compared with healthy tissues [35], confirming the involvement of the NLRP3 inflammasome in the pathogenesis of periodontitis. Bostanci et al. reported the biofilm-induced upregulation of inflammasome transcription causing early inflammation in periodontal tissues, whereas the downregulation of transcription enabled the survival of periodontal pathogens, thus proving that the biofilm can differentially modulate the activity of inflammasomes [86]. A study conducted in 2014 demonstrated a strong correlation between NLRP3 and advanced periodontitis [87]. According to Xue et al., the level of NRLP3 was higher in patients with chronic periodontitis or generalized aggressive periodontitis, compared to healthy subjects [80]. *Porphyromonas gingivalis* could activate inflammasomes through the involvement of gingipains [88].

### 2.4. Implications of the Inflammasome in General Pathologies

The inflammasomes also have an impact on systemic inflammation. Inflammasomes represent protein complexes located in the cytosol, which, through pathogenic stimulation, trigger inflammatory processes [89]. Inflammasomes cause the cleavage of pro-inflammatory cytokines, such as IL-1β and IL-18 [90]. The NLRP3 inflammasome reacts to a wide range of bacterial ligands, such as LPS, bacterial RNA and peptidoglycans. They are involved in the pathogenesis of periodontal disease, rheumatoid arthritis and osteomyelitis [63].

Caspase-1 is involved in the pathogenesis of various general pathologies such as Alzheimer’s disease, Crohn’s disease, rheumatoid arthritis, cardiovascular diseases and endometriosis [55], thus becoming a target for pharmaceutic agents.

According to a study conducted in 2015, NLRP3 and IL-1β are increased in subjects with chronic periodontal disease associated with type 2 diabetes compared to healthy subjects [91].

Inflammasome activation can be induced by ROS due to mitogen-activated protein kinases (MAPK). Numerous pathologies are characterized by increased ROS production, with indirect effects on inflammasome activation; these pathologies include viral infections, gout and diabetes [39]. NLRP3 inflammasome activation can also be induced without ROS generation or lysosomal damage [92]. The deficiency of 25-hydroxycholesterol, which is an inhibitor of cholesterol biosynthesis, triggers the release of mitochondrial DNA and activates inflammasomes and the secretion of IL-1 and IL-18 [93]. According to a recent study, cholesterol crystals can cause the release of NETs and the activation of the macrophage inflammasome [94].

Several studies have demonstrated the involvement of the NLRP3 inflammasome in the body’s immune responses to viral, fungal and parasitic attacks. Dengue is a viral infection caused by the *Dengue virus* (DENV), transmitted to humans through the bite of infected mosquitoes, leading to inflammasome activation, caspase-1 activation and IL-1β secretion [95]. Segovia et al. demonstrated a connection between the NLRP3 inflammasome and *respiratory syncytial virus* [96]. Additionally, caspase-1 and IL-1β secretion was activated by infection with *Mycobacterium tuberculosis* [97]. Another study demonstrated inflammasome activation by the crystalline compound alum, found in human vaccines [98]. The activation of inflammasomes was also observed in patients with diabetes [99], chronic kidney diseases [100] and autoimmune pathologies such as lupus erythematosus [101,102].

## 3. Production of Reactive Oxygen Species Mediated by Inflammasomes

The NLRP3 inflammasome senses membrane defects and induces an efflux of K^+^ ions, which generates mitochondrial ROS, which has a major role in inflammation [92]. 

Oxygen is a vital element for survival, but in high concentrations, it becomes toxic due to oxygen radicals, such as superoxide, hydroxyl, peroxyl and hydroperoxyl [103]. Through association with hydrogen peroxide and ozone, ROS are formed. The human body constantly produces ROS, but also reactive nitrogen species (RNS). According to current data, the following six reactive species are harmful to the human body: superoxide anion, hydrogen peroxide, peroxyl radicals, hydroxyl radical, singlet oxygen and peroxynitrite. Hydrogen peroxide is a reactive oxygen species which is continuously produced in most tissues of the body, the main producer being the mitochondria [104]. The human body fights against ROS and RNS through different antioxidants, which transform the reactive species into harmless compounds. Two main subtypes of antioxidants have been described: the preventive antioxidants, which are the first line of defense to stop the formation of reactive species, and the chain breakers, with a secondary role. Additionally, the tertiary line of defense removes detritus and restores functionality, and the quaternary line acts through adaptation mechanisms [105]. Vegetable intake decreases oxidative stress [106], whereas foods based on animal protein increase the risk of cardiovascular diseases [107]. Polyphenols have become the most important antioxidants [108]. 

In neutrophils, a chain of metabolic reactions is triggered, which activates protein kinase C and induces nicotinamide adenine dinucleotide phosphate (NADPH) oxidase to reduce oxygen and form superoxide radical, further metabolized to other ROS [81]. ROS are stored in phagosomes or released extracellularly, to act on pathogens that cannot be phagocytosed. Once the homeostatic balance is broken, neutrophils can induce lesions, which cause systemic inflammation [76].

**Figure 5:**
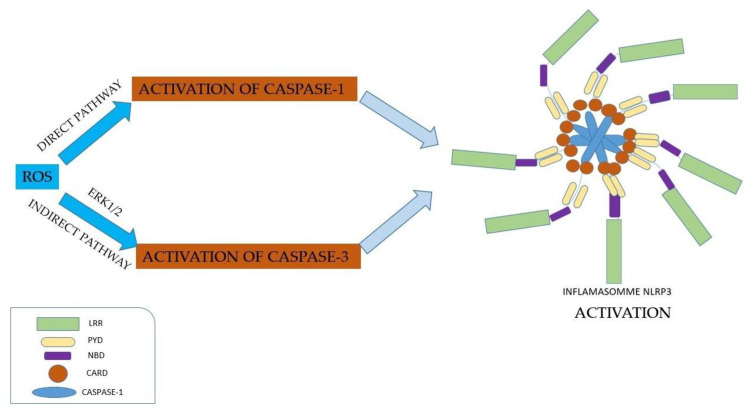
Graphical representation of mechanisms involving ROS production for direct activation of caspase-1 and indirect activation of caspase-3 activation through ERK1/2, which finally result in NLRP3 inflammasome activation (LRR—Leucine-rich-repeat, PYD—Domain Pyrin, NBD—Nucleotide-Binding-Domain, CARD—Caspase Activation and Recruitment Domain).

ROS can directly activate caspase-1; additionally, by an indirect mechanism involving ERK1/2, ROS activate caspase-3. ERK1/2 is implicated in inflammation by guiding cells to a proapoptotic state or an antiapoptotic state [39]. Kuiper indicated a model of NLRP3 inflammasome activation, through the APANAREI axis, which includes the P2X7 receptor, NOX, ROS, ERK1/2, NLRP3, and caspase-1. Thus, the APANAREI axis involves the activation of the P2X7 receptor, mediated by ATP, which induces the production of ROS, which in turn determines the activation of ERK1/2, then the activation of caspase-1 and, finally, the activation of the NLRP3 inflammasome [109] (Figure 5).

## 4. Inflammasome Inhibitors

### 4.1. Direct Inhibitors of the Inflammasome

Several pathogens can inhibit inflammasome activation, including Mycobacterium Tuberculosis, Yersinia specia, Legionella Pneumophila and Pseudomonas Aeruginosa [76], as well as Myxoma virus and Shope fibroma virus [110]. 

**Figure 6:**
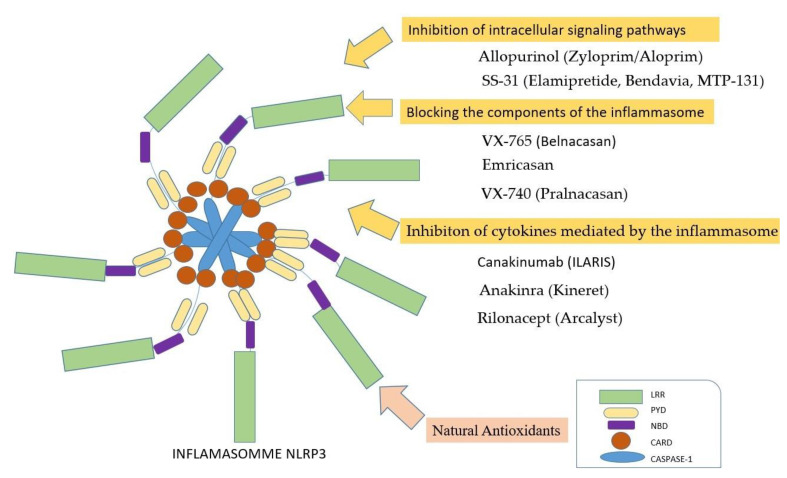
Therapeutic approaches for direct and indirect inflammasome inhibition. Direct inflammasome inhibition can be achieved by three methods: inhibition of intracellular signaling pathways, blockage of the inflammasome components, and inhibition of cytokines mediated by the inflammasome. The indirect inhibition can be achieved by pharmaceuticals that neutralize reactive oxygen species (ROS) and thus inhibit inflammasome activation. (LRR—Leucine-rich-repeat, PYD—Domain Pyrin, NBD—Nucleotide-Binding-Domain, CARD—Caspase Activation and Recruitment Domain).

Inflammasome inhibition can be achieved by three mechanisms (Figure 6): (i) the inhibition of intracellular signaling pathways; (ii) the blockage of inflammasome components; (iii) the inhibition of cytokines mediated by inflammasomes [36].

For the inhibition of signaling pathways, two pharmaceutical preparations can be mentioned: Allopurinol and SS-31. Allopurinol (Zyloprim/Aloprim^®^) was discovered in the 1940s in the Burroughs Wellcome program. This pharmaceutical preparation has been used for the treatment of gout and hyperuricemia, but also in type 2 diabetes [111]. SS-31 (Elamipretide, Bendavia, MTP-131) is a pharmaceutical preparation used to stabilize cardiolipin from the mitochondrial membrane; thus, the excessive production of ROS is prevented [112]. This pharmaceutical preparation has been used for the treatment of gout and hyperuricemia, but also in heart failure [113]. 

In order to block the inflammasome components, three pharmaceutical substances can be used: VX-765, Emricasan and VX-740. VX-765 is a reversible inhibitor of caspase-1 [114], used for the treatment of psoriasis and epilepsy [115]. Emricasan has possible indications in the treatment of diabetes and non-alcoholic hepatic steatosis [116]. The best preparation in this category is represented by VX-740 (Pralnacasan) [117]. Colchicine should also be mentioned; this pharmaceutical preparation has been used for over 3000 years for the treatment of gout [118], and is also indicated in heart diseases [119]. It has anti-inflammatory and antioxidant effects, but it is also a protector of bone structure [10]. The main mechanism of action is by blocking the assembly of microtubules [120]. 

Several methods have been described for inhibiting the cytokines mediated by inflammasomes. The inhibition of IL-1β can be achieved using a monoclonal antibody, Canakinumab, or an antagonist of the IL-1β receptor called Anakinra, and can also be achieved through a soluble receptor, Rilonacept [36]. These pharmaceutical preparations were used for the treatment of rheumatoid arthritis and diabetes [121,122]. Canakinumab (ILARIS) is indicated for the treatment of rheumatoid arthritis and atherosclerosis [123]. Rilonacept (Arcalyst) is used to treat Mediterranean fever [124]. Anakinra (Kineret) is indicated in the treatment of type 2 diabetes and Behcet’s disease [125].

### 4.2. Indirect Inhibitors of the Inflammasome by Acting on Reactive Oxygen Species

Several studies have demonstrated that plant antioxidants could counteract oxidative stress, through the redox properties of plant polyphenols acting as reducing agents and chelating metal ions [126]. Thus, it was suggested that phytochemicals may have beneficial effects [127], since ROS are neutralized by non-enzymatic antioxidants, such as vitamins C and E, but also by phytochemicals. 

*Aronia Melanocarpa* is a plant from the rosaceae family, which originates from North America and Russia, and its fruits contain high levels of flavonoids, including proanthocyanins, anthocyanins, flavonols and catechins, but also phenolic acids, which are hydroxylated derivatives from benzoic acid and cinnamic acid [128]. These fruits present a series of advantages due to their antidiabetic, antimutagenic and anticarcinogenic effects, as well as hepato- and cardio-protective properties [105]. In 2005, Ohgami et al. demonstrated the anti-inflammatory effect of *Aronia Melanocarpa* extract, suggesting that 100 mg of extract had similar effects to 10 mg of prednisolone [129]. 

According to Tauber et al., flavonoids controlled the production and release of ROS by directly scavenging reactive oxygen [130]. Some flavonoids can scavenge hypochlorous acid and reactive chlorinated species, whereas other flavonoids can suppress the release of arachidonic acid from cell membranes [131]. Flavonoids can also inhibit cyclooxygenase and lipoxygenase and reduce the release of hydrolytic enzymes from lysosomes, according to the study of Tordera et al. [132]. In 1998, Middleton demonstrated that flavonoids can inhibit protein kinases [131]. Moreover, antioxidants could be used for limiting inflammasome activation [39] (Figure 6). 

Studies have shown that by preventing ROS formation, the production of IL-1β can also be inhibited; however, no influence on IL-18 production has been observed [133].

According to the current literature, curcumin could inactivate the NLRP3 inflammasome, thus exerting an anti-inflammatory effect. This phytochemical agent could be included in the category of indirect inflammasome inhibitors, along with flavonoids [134].

## 5. Biomaterials Functionalized with Inflammasome Inhibitors

Although nanobiomaterials are intensively studied in various medical and related fields, the transfer of this information to the usual practical applicability has not yet been achieved. These can present a series of benefits and advantages that improve the quality of treatments, but also a series of limitations and adverse effects that have not been sufficiently studied in the literature. By studying the physical–chemical properties of the various pharmaceutical preparations, various ways of incorporating them into specialized matrices could be discussed for the easier treatment of the various pathologies of the oral cavity. According to the specialized literature, there have been recently published studies that deal with these topics and that can be helpful in discovering various pathologies or even in therapeutic implications. The detailed study of these diagnostic or therapeutic options, even if at this moment it represents a new challenge for researchers, may be a track for future possible research, which will bring new information to the scientific community and benefits for different pathologies [135]. 

In periodontology, biomaterials are used to replace bone or soft tissue defects. Periodontal therapy uses allogeneic or autologous grafts, which represent the golden standard up until this point [135]. Even if this therapeutic option is considered a “golden standard”, its limitations are numerous, the most important being represented by the need for donors, possible immunological incompatibilities, their limited quantity and especially the possibility of treating different infectious diseases. For a material to be considered ideal, it must have a series of properties: the most important is biocompatibility, followed by elasticity, mechanical strength, ductility and durability, malleability and good thermal properties [136]. 

Nanomaterials, through their optical, mechanical, electronic and magnetic properties, have gained a special place in therapeutic approaches [137]. Moreover, the possibility of using different types of nanomaterials, such as nanoparticles, nanotubes, nanocapsules, nanocomposites and nanofibers, to restore tissue functionality has been discussed [138].

Periodontal disease therapy has two major stages, the first being represented by non-surgical periodontal therapy (professional mechanical instrumentation, bacterial plaque control, local antimicrobial adjuvant treatment, the elimination of local risk factors and the management of systemic risk factors), and the second being represented by surgical periodontal therapy. Surgical periodontal therapy is a complex approach, which encompasses three broad types of therapeutic possibilities, these being the following in the beginning: bone grafts [139], guided tissue regeneration [140] and the use of plasma rich in platelets, known as PRP [141], all with the aim of reducing inflammation and restoring optimal functionality. Nanomaterials with dimensions below 100 nm have recently been introduced, presenting a series of advantages, such as versatility, biocompatibility, the controlled release of pharmaceutical substances and mechanical resistance. According to a recent literature review, the most researched materials for bone regeneration were represented by nanohydroxyapatite and collagen. Both materials present excellent osteoconductive, mechanical and biological properties [142]. Polylactic glycolic acid and polycaprolactone are intensively studied for use in tissue engineering and regenerative medicine due to their ability to degrade into compounds that can be easily metabolized and excreted. According to studies using these biomaterials for drug delivery, they can provide long-term effects and prevent inflammation [143]. It is also known that polylactoglycolic acid must be reinforced with ceramic nanoparticles to provide adequate resistance to various therapeutic procedures [144]. 

The structures on which pharmaceutical substances that act by inhibiting the inflammasome, and thus reduce the level of inflammation, are adsorbed should be based on a conductive material. In order to ensure the controlled release and possibly the slow release of the adsorbed preparations, polymers can be used.

Nanofibers have also been studied for their ability to transport pharmaceutical substances. According to a study carried out in 2022, they were prepared by electrospinning, forming a polylactic acid-hydroxyapatite-doxycycline (PLA-HAP-Doxy) system. According to this study, the nanofibers were prepared in two ways: immobilization on the surface and encapsulation in the fiber structure.

It was demonstrated that the polylactic acid–hydroxyapatite–doxycycline system can be used for biomedical applications [145]. 

Electrospun nanofibers are indicated for the application of biomedical substances, due to their property of being flexible and their high production rate [146]. Other useful properties of these fibers are represented by their large surface area, the variable degree of porosity and controllable morphology, which gives the medicinal substance an increased bioavailability [147]. The design method of the nanofiber–drug system depends on the nature and characteristics of the pharmaceutical substance which is desired to be adsorbed on the surface of the nanofiber. It is known that the release of the active substance depends on the interaction between the polymer and the pharmaceutical substance, the diameter of the fiber and the initial content of the substance [145]. A series of polymers and combinations of polymers can be used to control the release of the adsorbed substance, such as polylactic acid, polyethylene-vinyl acetate and polycaprolactone [148]. Nanofibers with hydroxyapatite and polylactic acid systems have been shown to have high biocompatibility [149] and good tissue response [150,151]. 

The biomaterial based on polylactic acid, nanohydroxyapatite and doxycycline, according to a study published in 2023, proved to have beneficial effect for the treatment of periodontal disease, decreasing the clinical parameters of inflammation, but also influenced the salivary biomarkers, by decreasing the salivary concentration of MMP-8, IL-1 and TNF-α [152]. Due to the influence on biomarkers, the biomaterial was considered to have a local and systemic biomodulatory effect in periodontal disease. Nanofibers containing polylactic acid have an increased mechanical resistance to stress, a slow degradation rate and the possibility of degradation without releasing toxic residues [153].

Pharmaceutical and phytochemical substances mentioned in the specialized literature as possible inhibitors of inflammasomes could be investigated in future studies in terms of their adsorption on different nanostructures, so that they can be used for topical applications in oral therapy. In order to be able to establish a connection between the pharmaceutical substance, the nanostructure on which it will be incorporated and the ligand that will be able to control the release of the preparation, the physicochemical properties of these substances must first be studied. Biomaterials can block inflammasome activation through several mechanisms: (i) the inhibition of intracellular signaling pathways, (ii) blocking the components of the inflammasome, (iii) the inhibition of cytokines mediated by the inflammasome, (iv) the inhibition of ROS (Table 1). 

### 5.1. Blocking of the Inflammasome by Inhibiting Intracellular Signaling Pathways

For the inhibition of inflammasomes by inhibiting intracellular signaling pathways, two pharmaceutical substances have been mentioned: Allopurinol (Zyloprim/Aloprim^®^) and SS-31 (Elamipretide, Bendavia, MTP-131). Allopurinol, also known as 1,2-dihydro-4H-pyrazolo [3,4-d] pyrimidin-4-one, is an odorless, tasteless white microcrystalline powder. Allopurinol is a bicyclic organic compound in which a pyrazole ring is linked to a hydro-substituted pyrimidine ring. It is considered to be a structural analogue of hypoxanthine, a natural purine base. In terms of its application, it is often used in the treatment of gout and hyperuricemia as it inhibits the action of the xanthine oxidase enzyme [154,155]. The main disadvantage of the drug is that it is poorly soluble in water (0.1 mg/mL) [156]. Changdeo et al. prepared solid dispersions of allopurinol using different methods and hydrophilic carriers such as polyvinylpyrrolidone, polyethylene glycol 6000 to increase the water solubility. The presence of polymers had a positive effect on increasing the water solubility [156]. Kandav et al. prepared chitosan nanoparticles loaded with allopurinol to improve solubility and bioavailability [157]. Varrica et al. have incorporated allopurinol into nanostructured lipid carriers (NLCs) in order to improve skin penetration, solubility problems and therapeutic index of the drug. The nanostructured lipid carriers are able to make allopurinol soluble and to incorporate and deliver it into the skin [158]. Kandav et al. have produced bovine serum albumin nanoparticles loaded with allopurinol in order to deliver allopurinol to the kidney [159]. According to the literature, it can be seen that various methods have been attempted to incorporate Allopurinol into various nanostructures.

SS-31 (Bendavia) is a small, easily synthesized cell-permeable peptide that is used in therapy because of its water solubility. It is a variable aromatic, cationic tetrapeptide with structural properties that targets the mitochondria, penetrating its inner membrane, associating with cardiolipin and restoring membrane stability [160]. To efficiently deliver SS-31, Di Liu et al. designed nanopolyplexes complexed using anionic hyaluronic acid and cationic chitosan. The electrostatic equilibrium of the nanoparticles is disrupted in the low pH environment of lysosomes, thus allowing SS-31 to be released and exert its therapeutic effect in the mitochondria. Studies have shown that the nanoparticles exhibited superior therapeutic effects compared to free SS-31 [161]. Due to the increased solubility in water, this pharmaceutical preparation is useful in therapy, and according to the study conducted by Di Liu et al., its ability to be released from the structure on which it is adsorbed and the ability to exert its therapeutic effect at the site of action has been confirmed. 

### 5.2. Inhibition of the Inflammasome by Blocking Its Components

For the inhibition of inflammasomes by blocking the components of the inflammasome, three pharmaceutical substances were mentioned in the specialized literature: VX-765, Emricasanul, VX-740 (Pralnacasan). The action of colchicine should also be mentioned. 

Belnacasan, also known as VX-765, acts as a potent and selective inhibitor of the caspase-1 enzyme, which is being investigated for the treatment of epilepsy. It is an orally active prodrug that is converted to the VRT-043198 active drug in the body. In physical appearance, it is a light yellow powder, insoluble in water. 

Emricasan, also known as IDN-6556, is a white, solid powder that is insoluble in water but not in DMSO. Its molecular structure is C_26_H_27_F_4_N_3_O_7_ and its molecular weight is 569.50 g/mol [162]. Emricasan belongs to the caspase family and is an irreversible pan-caspase inhibitor. It is used in patients with non-alcoholic steatohepatitis because it reduces serum aminotransferases and caspase activity. It was the first caspase inhibitor to be tested in humans [163].

Pralnacasan, also known as VX-740, is a peptidomimetic caspase-1 inhibitor targeting inflammatory components. It is used in clinical trials as a potent non-peptide inhibitor of the interleukin 1β converting enzyme [164]. Pralnacasan has a 2-naphthyl hydrophobic moiety at the P4 position and a pyridazinodiazepine-based bicyclic core that mimics the Val-Ala region. A lactone moiety is located at the C- terminal, covering an aspartate residue at the P1 position. Since the discovery of the drug, researchers have sought to produce a number of inhibitors with a mono- or bicyclic backbone mimicking P3-P2 [165].

Colchicine is a light yellowish powder with the molecular structure C_22_H_25_NO_6_ [166]. It is an alkaloid that contains an N atom in the amide form. Due to its toxic properties, its use as a medicine has been marginalized and it is often identified as a mitotic damager [167]. Colchicine is a compound containing three rings (A, B, C), in which ring A is a trimethoxy phenyl ring, ring B is a saturated seven-membered ring and ring C is a tropnol ring. Colchicine is not a basic compound and therefore, unlike other basic alkaloids, it does not form salts. It forms precipitates with various alkaloid reagents. Colchicine has a chiral center at the C-7 position. Due to the presence of a chirality axis, Colchicine is asymmetric, with the A ring twisted by 53° relative to the B ring. Colchicine has four enantiomers; however, it should be emphasized that this requires the (aS) configuration for binding to tubulin [168]. Colchicine is an ancient drug, mentioned in the writings of several Greek physicians such as Nicader of Colphan, Discorides and Alexander of Trolles. It has also been used to relieve gout-induced pain and to treat an autoinflammatory genetic disorder, none other than familial Mediterranean fever. According to the specialized literature, these pharmaceutical preparations have a possible action on the inhibition of inflammasomes, and through the detailed study of their physicochemical properties, nanobiomaterials could be discovered on which they can be incorporated, to be used in the treatment of oral pathologies.

### 5.3. Blocking of the Inflammasome by Inhibiting the Cytokines

For the inhibition of inflammasomes by inhibiting the cytokines mediated by the inflammasome, three pharmaceutical substances have been mentioned in the specialized literature: Canakinumab, Anakinra and Rilonacept. 

Canakinumab, also known as INN, is a human anti-IL-1β monoclonal antibody with high affinity. Canakinumab consists of two light chains of 214 residues and two heavy chains of 447 or 448 residues. It has a molecular mass of 145 kDa. The molecular formula of canakinumab is based on the amino acid composition without post-translational glycosylation, but includes N-terminal pyroglutamate formation and lysine residues on the C-terminals of the heavy chains [169]. 

Anakinra is a 153-amino-acid recombinant human interleukin-1 (IL-1) receptor antagonist. Anakinra differs from native human IL-1Ra in that it has a methionine group at the amino terminal [170]. 

Rilonacept is a dimer fusion protein, an engineered compound consisting of the extracellular domains of 1L-1-RacP and 1I-1-R1. Also known as Il-1 trap, it is linked to the Fc portion of human immunoglobulin G1 (IgG1) [171].

Monoclonal antibodies could be used in conjunction with anti-angiogenic therapy as a strategy to improve the outcome of cancer immunotherapy, as described by Li et al. Thus, it has been shown that Ipilimumab exerts the direct control of immune reactions by regulating the activation and proliferation of T cells. Due to intra-tumoral hypoxia, the activity of T cells is inhibited, leading to the alteration of the adaptive immune response. According to the literature, hypoxia seems to modulate the immune response; however, the current data are insufficient, requiring additional research to understand the complex mechanisms underlying the influence of hypoxia on immunotherapy. Another factor that could influence the immune response is the acidic environment, which alters cytokine production and reduces the activation and priming of T cells [172].

### 5.4. Inhibition of the Inflammasome by Using Phytochemicals

Phytochemicals can also act on inflammasomes in order to inhibit their action through the increased content of flavonoids. Flavonoids are a family of compounds that are widely distributed in the plant kingdom and have many medicinal properties. They belong to the group of polyphenols and are found in many plants (chamomile), fruits (oranges and grapefruit) and vegetables (onions and broccoli), as well as in beverages. They are structurally characterized by a 2-phenylchroman skeleton (C6-C3-C6) with a heterocyclic pyrene ring fused to the benzene ring labelled A and linked to the phenyl ring labelled B. The flavonoid structures known to date have various substituents such as multiple hydroxyl (-OH), methoxyl (-OCH3) and glycoside groups, as well as an oxo group located at position 4 of the C ring [173,174]. Within the flavonoid family of compounds, there are different subgroups. These subgroups depend on the degree of oxidation, saturation, substitution pattern and bonding position of the C ring to the C2/C3 and C4 carbon atoms of the B and C rings. For example, in the case of isoflavones, the B ring is attached to the triple position of the C ring, whereas in the case of neoflavonoids, the B ring is attached to the quadruple position of the C ring. The metabolism, biochemical and pharmacological activity and bioavailability of flavonoids depend on the structural characteristics and configurations of the flavonoids [175]. These compounds have unique chemical and physical properties. These properties affect the solubility of flavonoids in different solvents. Their solubility in water is affected by the presence of a double bond in the C ring and the number of hydroxyl (-OH) substituents in the B ring. The solubility in water increases with the number of hydroxyl groups, whereas the solubility in 1-octanol is adversely affected by the presence of hydroxyl groups. The stability of flavonoids changes when they are exposed to external stimuli such as heat and light. The scientific literature indicates that flavonoids undergo oxidative transformation when exposed to the aforementioned effects [175].

Over the past few decades, the development of targeted drug delivery tools has undergone intensive development to meet stringent requirements. These requirements include the need for a delivery system to increase the effectiveness of the treatment, while at the same time reducing undesirable effects. Importantly, the drug delivery system must be biocompatible and not form toxic compounds. The scientific literature shows that over the past few decades, researchers have succeeded in developing drug carriers with a wide range of chemical structures and origins, ranging in molecular sizes from 1 to hundreds of nanometers. Second-generation carriers smaller than 1 μm, belonging to the group of so-called colloidal carriers, are used to deliver flavonoids. The main nanocarriers suitable for the delivery of flavonoids include phytosomes, liposomes, solid lipid nanoparticles (SLN), nanostructured lipid carriers, nano- and microemulsions, polymer nanoparticles, chitosan based nanoparticles, gold-based nanoparticles, silver-based nanoparticles and silica nanoparticles. Zverev et al. have shown that phytosomes containing flavonoids are characterized by a long-lasting tumor-targeting effect. The main property of this drug delivery system is that it does not exert toxic effects on the surrounding healthy cells and tissues. The aforementioned Zverev et al. reported that liposomes loaded with a flavone called luteolin and coated with tocoferyl polyethylene glycol succinate (TPGS) had no adverse effects on healthy tissues, but showed increased cytotoxicity in human lung cancer cells [176]. 

## 6. Discussion

Inflammasomes are part of the innate immune system, which normally acts beneficially on the body, but their uncontrolled activity causes harmful effects, being able to induce autoinflammatory or autoimmune diseases [177]. According to the current data, therapeutic action on inflammasomes could be considered a new treatment option for periodontal disease, as it decreases the production of cytokines [78]. 

Some pharmaceutical preparations and phytochemical preparations can cause the inhibition of the inflammasomes. In order to facilitate the therapeutic effects, the adsorption or inclusion of these preparations on various structures of nanobiomaterials could be discussed. For use on a large scale and to facilitate various treatments, these nanobiomaterials should be involved in several studies in order to be able to exclude their limitations: inducing immunological reactions, spreading in unwanted sites, the occurrence of cytotoxicity and the control of biodegradation. According to studies, the biodistribution and pharmacokinetics of these preparations depend on their shape and size [178]. Even if the incorporation of pharmaceutical preparations on various biocompatible structures could be of great help in therapy, their limits, their biocompatibility and the way they will interact with the human body must be studied. 

According to a literature review, it can be stated that the major problem with these materials is related to toxicity. They can enter the circulatory system and induce hematological toxicity, they can accumulate in organs (lungs, liver, spleen, kidneys) or they can produce an exaggerated quantity of ROS [179]. It is known that the size of nanomaterials is the physicochemical property that majorly influences the cellular response [180]. A nanomaterial with a reduced size presents an inversely proportional surface–volume ratio, which determines their greater reactivity [181], but also a lower accumulation in the liver and spleen [182]. However, having a small size, nanoparticles can cross the blood–brain barrier, thus presenting major health risks. The shape of the material influences the mode of absorption, biodistribution and their use [180]. It is known from the literature that a spherical shape of biomaterials has a superior capacity of pharmaceutical substance delivery than other shapes of biomaterials [183]. The asymmetric shapes of nanomaterials have an increased capacity to penetrate tissues and release pharmaceutical preparations [184]. It is also known that intelligent particles are able to change their shape depending on the needs of the site [185]. Another set of properties that influence their therapeutic potential is represented by the loading capacity, binding capacity and hydrophobicity [180]. Thus, surfaces with positive charges have an increased absorption, and those with negative charges have a greater capacity to circulate for a longer time [186]. 

Current research has focused on the encapsulation of drugs into nanoparticles in order to treat autoimmune diseases and allergies by inhibiting the immune response. The limitations of this technique are related to the low encapsulation capacity derived from hydrophobic property of the drugs. By using mRNA lipid nanoparticle (LNP) platforms, the disadvantage related to hydrophobicity could be overcome, and nanoparticles**’** loading with different therapeutic substances could be improved. Yim et al. demonstrated that through using this approach for drug administration, both cellular and subcellular levels could be targeted. Another limitation related to nanoparticles is the complex synthesis process. Therefore, the current research in the field of nanotechnology aims at increasing therapeutic efficiency and reducing the risk of side effects. In addition, efforts are made to optimize the biological properties of matrices designed for loading the active substances, in order to ensure the most targeted therapeutic effects [187].

## 7. Conclusions

Due to the possibilities of the adsorption on the surface or inclusion in nanofibers of various pharmaceutical preparations, this newly exploited branch of the universe of materials can represent a promising perspective for clinical practice, helping practitioners to improve the quality of life of patients. Thus, these new nanobiomaterials and their combinations deserve to be studied and researched with interest, due to their capacity of action on the human body.

## Figures and Tables

**Table 1:** Therapeutic approaches for inhibiting the inflammasome, by direct and indirect mechanisms.

Type of Inhibition	Mechanism of Action	Therapeutic Agents
Direct	Inhibition of intracellular signaling pathways	- Allopurinol (Zyloprim/Aloprim^®^) - SS-31 (Bendavia)
Direct	Blockage of inflammasome components	- VX-765(Belnacasan) - Emricasan - VX-740 (Pralnacasan)
Direct	Inhibition of cytokines mediated by inflammasome	- Anakinra - Canakinumab - Rilonacept
Indirect	Inhibition of inflammasome by acting on reactive oxygen species	- Flavonoids

## Data Availability

Not applicable.
